# Case Report: False aneurysm as a late unusual complication of the aortofemoral bypass graft in a patient with critical leg ischemic symptoms: interesting case

**DOI:** 10.3389/fradi.2024.1327050

**Published:** 2024-05-01

**Authors:** M. P. Belfiore, R. Zeccolini, P. Roccatagliata, L. Gallo, A. Fabozzi, S. Cappabianca

**Affiliations:** Department of Precision Medicine, University of Campania “Luigi Vanvitelli”, Naples, Italy

**Keywords:** pseudoaneurysm, Computed Tomography-angiography, aortofemoral bypass surgery, angiography, lower extremity ischemic symptoms

## Abstract

Aortofemoral bypass surgery is a common procedure for treating aortoiliac occlusive disease, also known as Leriche syndrome, which can cause lower extremity ischemic symptoms. Diagnostic imaging techniques play a crucial role in managing pseudoaneurysms (PSAs), with Duplex ultrasound and Computed Tomography-angiography (CTA) being effective tools for early diagnosis. Pseudoaneurysms (PSAs) present as pulsating masses with various symptoms, and prompt intervention is essential to avoid complications. A case report is presented involving an 82-year-old male who underwent aorto-bifemoral bypass surgery and later developed a pseudoaneurysm (PSA) of the left branch. Surgical treatment involved the removal of the pseudoaneurysm (PSA) and graft replacement. Other cases from the literature are also described, emphasizing the rarity and potential severity of non-anastomotic pseudoaneurysms (PSAs) in reconstructive vascular surgery. Periodic screening of patients who undergo reconstructive vascular surgery is crucial to detect pseudoaneurysms (PSAs) early and prevent complications. Asymptomatic pseudoaneurysms (PSAs) can grow significantly and become life-threatening if not identified in a timely manner. Regular post-operative imaging, such as annual Computed Tomography-angiography (CTA) and/or Duplex ultrasound, is recommended to ensure early diagnosis and appropriate management of complications.

## Introduction

Aortofemoral bypass surgery is a commonly used procedure for treating aortoiliac occlusive disease, also known as Leriche syndrome. This condition can cause lower extremity ischemic symptoms, necessitating medical intervention such as claudication, rest pain in the lower extremities, or the development of ischemic ulcers due to insufficient blood flow, but some patients may not show any symptoms. If endovascular techniques are unsuccessful or not appropriate, aortobifemoral bypass remains a crucial option and is even considered the gold standard for achieving long-term patency. The diagnostic gold standard method for aortoiliac occlusive disease is the Computed Tomography-angiography (CTA). Pseudoaneurysms (PSAs) are considered a late complication of prosthetic reconstructive vascular surgery, and are commonly detected at the level of anasthomotic sites. In most cases, they are encountered by chance, and are recognized only when they reach considerable size as a pulsating mass. Associated signs may involve ecchymosis, erythema, and tenderness upon palpation. Complications associated with pseudoaneurysms (PSA) enclude rupture, distal embolization, local pain, neuropathy, and skin ischemia in the affected area. Regular post—operative follow-up is essential to early detect potential further enlargement or rupture, which could lead to the necessitation of extensive surgical procedures. The case we discuss is of an 82-year-old man who came to our observation with the evidence of a pulsating mass at the level of the left iliac region. Patient overlooked reporting previous trauma only citing history of aorto-bifemoral bypass prosthetic graft surgery about 10 years earlier undergone to treat chronic obstructive arteropathy of the lower extremities (Table 1). Subsequently, the Duplex Ultrasound and the Computed Tomography-angiography (CTA) detected the presence of a voluminous non-anastomotic pseudoaneurysm (PSA) (1).

**Table 1:** The table describes the instrumental tests performed by the patient.

Sex	Male
Age	82
Symptoms/Signs	Pulsatile mass and weight sensation in left iliac region.
First-line diagnostic exam	*Ultrasound*: suspected the presence of a voluminous hematoma next to the left branch of the aorto-bifemoral bypass.
Second-line diagnostic exam	*CTA:* detected the presence of a pseudoaneurysm at the left branch of the aorto-bifemoral bypass.
Intervention	Resection of the PSA and reconstruction with a prosthetic implant, followed by a femoral deep right cross-over retro-pubic bypass.

## Case-report

**Figure 1:**
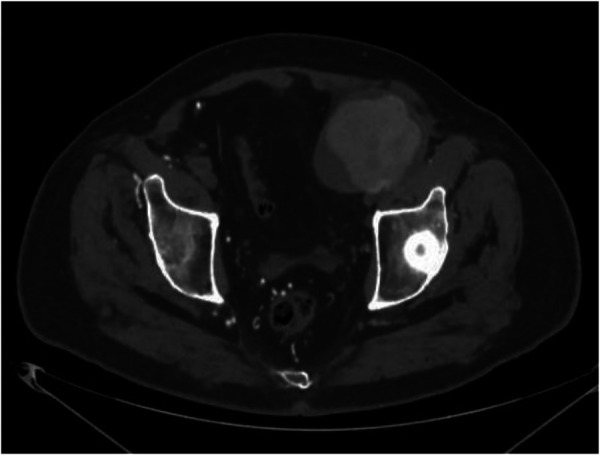
Computed Tomography-angiography axial scan: voluminous pseudoaneurysm at the level of the left branch of the bifemoral aorto bypass.

**Figure 2:**
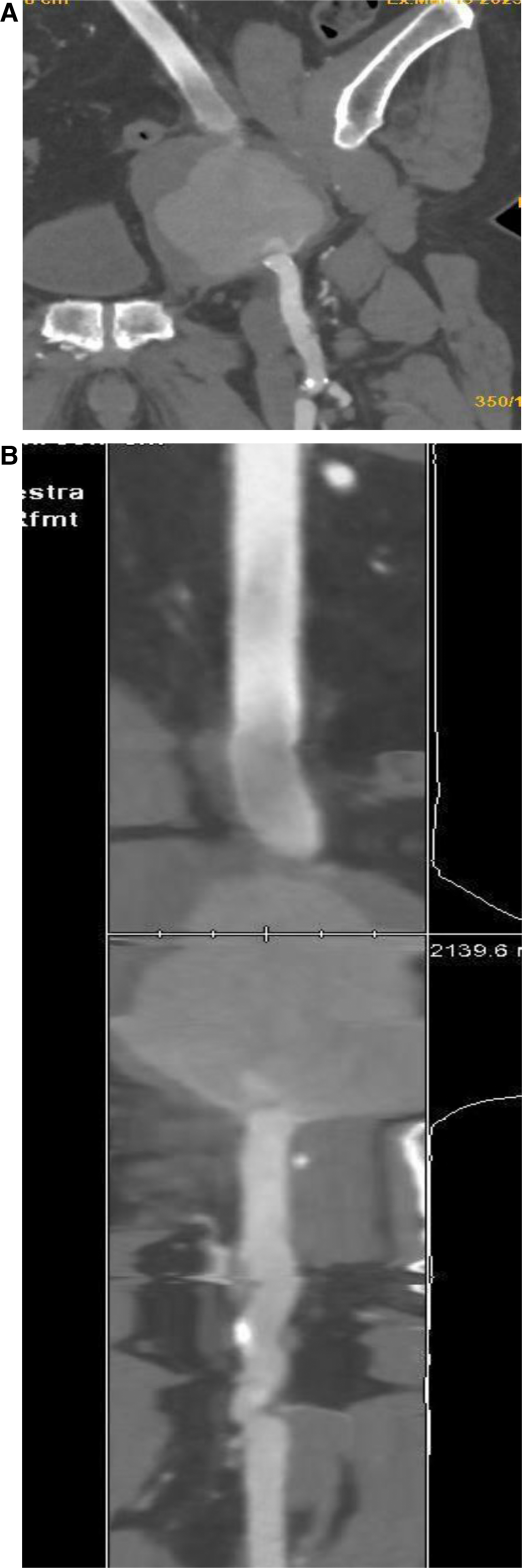
Computed tomography 3D multiplanar reconstruction (**A**) describes the presence of pseudoaneurysm at the level of the left branch of the bifemoral aorto-by-pass (**B**) better displayed in the coronal view (**B**).

**Figure 3:**
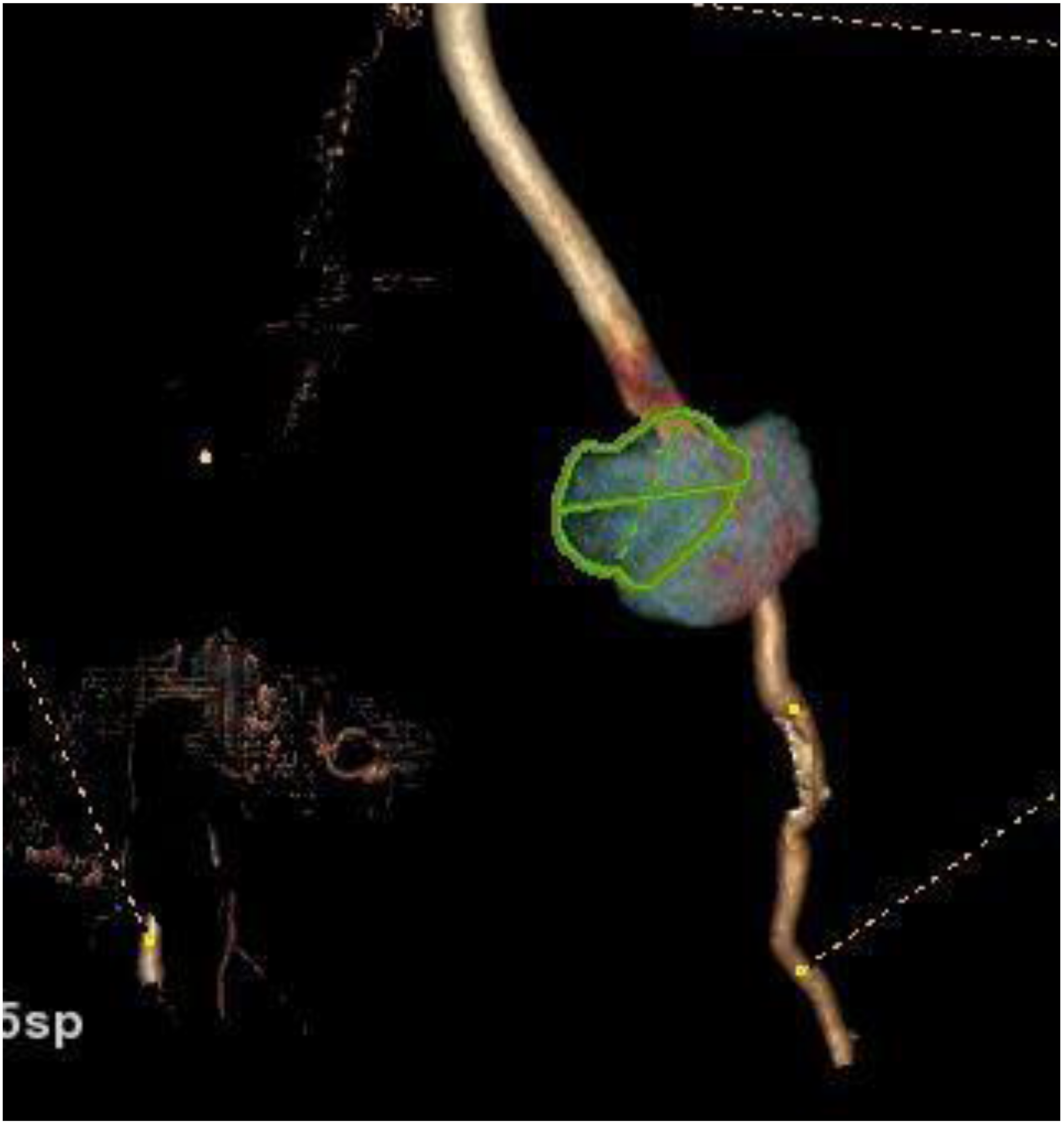
3D reconstruction CT.

Our 82-year-old male patient presented with pulsatile mass and sensation of heaviness in the left iliac region, along with intermittent claudication. The very first US examination performed in a different center suggested the presence of a voluminous hematoma right next to the left branch of the aorto-bifemoral bypass. Subsequently, a high-flow Computed Tomography-angiography (CTA) confirmed the presence of a pseudoaneurysm (PSA) of the left branch of the aorto-bifemoral bypass, situated approximately 12 cm from the bisiliac bifurcation. Additionally, the CTA revealed an occlusion of the right graft limb as well. Native common iliac, internal iliac, and external iliac arteries were found to be occluded on both sides. However, the popliteal and anterior tibial arteries, along with the tibioperoneal trunk, displayed normal opacification, course, and size. There were no indications of graft infection, such as perigraft air or fluid, and soft-tissue changes were absent (Figures 1–3). Furthermore, no traumatic injuries were detected in the abdominal organ parenchyma or in any of the examined bone segments (1).

**Figure 4:**
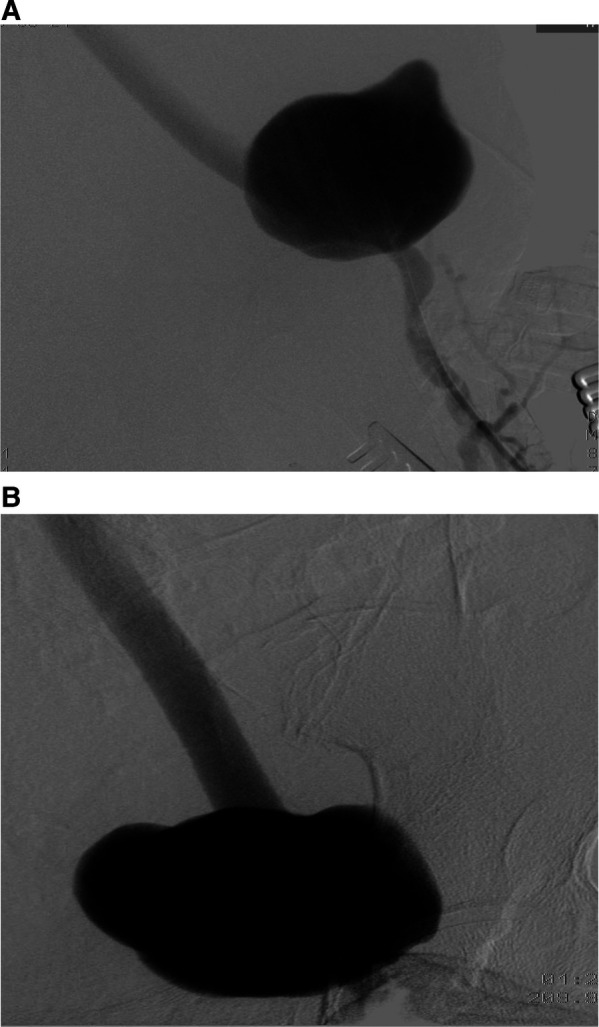
Preoperative aortofemoral angiography images (**A**,**B**).

**Figure 5:**
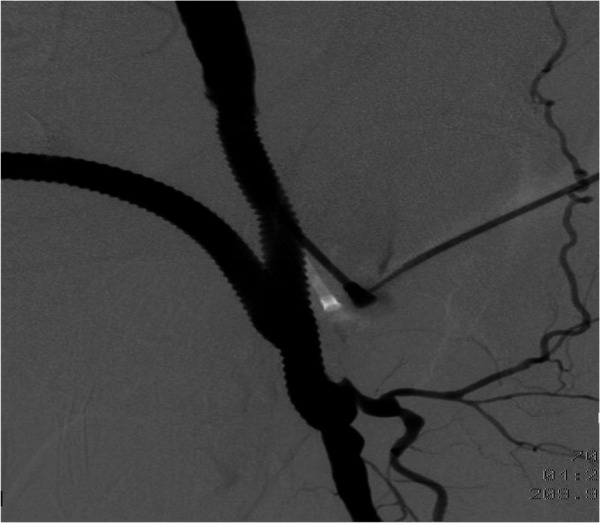
Postoperative aortofemoral angiography confirms complete excision of the left femoral pseudoaneurysm and its replacement with a prosthetic graft.

The patient was transferred to the vascular surgery department for treatment. The procedure involved the resection of the false left femoral aneurysm using a prosthetic implant, followed by a femoral deep right cross-over retro-pubic bypass. Throughout the treatment, no complications arose. Pre-operative and post-operative control angiography were conducted to assess the results (Figures 4, 5).

The patient's postoperative recovery was smooth, and they were discharged from the hospital on the fourth day without any issues.

## Discussion

False aneurysms, also called pseudoaneurysm (PSA), are typically described as abnormal enlargements of arteries restricted only by the tunica adventitia. However, it is important to view pseudoaneurysms (PSAs) as extravascular hematomas confined by reactive connective tissue. The hematoma surrounding the vessel, known as the “sac,” is in communication with the vessel lumen through a small collar called the “neck,” which permits the flow of blood from the vessel lumen into the hematoma (2) Numerous risk factors associated with the development of pseudoaneurysms (PSAs) have been identified, including female sex, hypertension, smoking etc.

Additionally, chronic obstructive pulmonary disease (COPD) is also recognized as a risk factor due to its impact on elastosis and degeneration of the vessel wall's connective tissue. Furthermore, COPD may contribute to diffuse atherosclerosis, leading to the formation of plaques that can erode the inner lamina and ultimately result in the formation of PSA (2, 3).

Pseudoaneurysms (PSAs) typically manifest as a pulsating mass accompanied by ecchymosis, erythema, and tenderness upon palpation. In cases where an infection is present, additional signs such as nonspecific symptoms like warm and reddened skin, malaise, back pain, fever, gastrointestinal bleeding, elevated sedimentation rate, hydronephrosis, or ischemia caused by a clot may be observed (4, 5). Pseudoaneurysms (PSAs) commonly arise as complications of catheter-associated interventional procedures. The incidence rate is approximately 0.06%–0.18% after diagnostic procedures but increases to 0.7%–6.25% after therapeutic interventions (3).

Likewise, it is regarded as a delayed complication of prosthetic reconstructive vascular surgery, occurring at an incidence rate ranging from 2% to 29%. While anastomotic pseudoaneurysm (PSA) is a well-known complication, non-anastomotic localization is exceptionally rare and uncommon (4, 6). Various factors contributing to the formation of pseudoaneurysms (PSAs) on prosthetic graft bypass have been documented and theorized in the literature. Among these, the most prevalent causes seem to be trauma, biodegradation of prosthesis fibers, mechanical stress, graft fabrication defects, tear due to damage during implantation, and repetitive hydrodynamic microtrauma (6–11).

The review conducted by Van Damme et al. highlighted several reasons for the intrinsic structural failure of dacron grafts, particularly in vulnerable areas such as the remeshing line or the black guideline. Other reported causes include repetitive hydrodynamic microtrauma resulting from pulsatile and turbulent blood flow, mechanical stress during fabrication and after implantation, and additional external forces like compression and stretching of the prosthetic branch of a bifurcated graft under the inguinal ligament. Biodegradation of the polymer, mishandling of the graft, and manufacturing defects, such as excessive heating during yarn texturization and crimping by thermo—fixation, excessive stretching of yarns during knitting, and chemical cleaning, as well as gamma or beta ray sterilization, were also identified as contributing factors to graft failure.

Diagnostic imaging techniques play a critical role in the management of pseudoaneurysms (PSAs), facilitating early detection and timely intervention. Duplex ultrasound has demonstrated high sensitivity and specificity, with values of 94%–99% and 94%–97%, respectively, in diagnosing a PSA. Meanwhile, Computed Tomography-angiography (CTA) exhibits specificity and sensitivity ranging between 95.1% and 98.7%, further contributing to accurate diagnosis and assessment of pseudoaneurysms (PSAs) (3). Grayscale ultrasound reveals a sac-like structure with hypo/anechoic appearance, linked to the vessel lumen through a hypo/anechoic neck. Within the pseudoaneurysm (PSA), a thrombus with varying echogenicity may be observed, either surrounding it or within the lumen. When using Duplex ultrasound, a distinct feature known as the “yin and yang sign” becomes evident, characterized by swirling and bidirectional systolic and diastolic blood flow in and out of the pseudoaneurysm (PSA) (3) Instead Computed Tomography-angiography (CTA) shows a round or lobulated contrast-filled saccular structure, similar in attenuation to adjacent artery, surrounded by non-enhancing hyperdense fluid representing hemorrhage or hematoma. The finding on CT of perigraft air and/or fluid, as well as soft-tissue attenuation, can be considered a sign of graft infection, and thus the cause of pseudoaneurysm (PSA) formation.

In general, when PSA convolves peripheral arteries, ultrasonography allows accurate diagnosis, whereas in cases of suspected psedoaneurysm (PSA) in the aorto-iliac site, Computed Tomography—angiography (CTA) is preferable.

In our case-report, the patient came to our observation with a pulsating mass located in the left iliac fossa, extending to the crural arch. The patient had undergone aorto-bifemoral bypass prosthetic graft surgery about 10 years earlier due to chronic peripheral obstructive arteriopathy of the lower extremities resulting in claudication. There were no signs of infection, and no prior direct trauma had been documented. Computed Tomography-angiography (CTA) revealed a voluminous 9 cm diameter pseudoaneurysm (PSA) at the left branch of the bypass, located at the level of the crural arch a short distance from the distal anastomosis. The right branch of the bypass was completely occluded.

As a result, our patient underwent a subsequent surgical procedure, involving the excision of the left femoral pseudoaneurysm (PSA) and its replacement with a prosthetic graft. Additionally, a femoral—femoral cross-over bypass was performed during the same surgery.

Unfortunately, we did not send the excised, old Dacron graft to microscopic or electron microscopic examination to investigate the real cause of graft disruption. As there was no clear information regarding the patient's medical history, and there were no signs of infection or outcomes of previous trauma, we hypothesized that the most likely cause of graft destruction resulting in pseudoaneurysm (PSA) formation was intrinsic structural failure of a fabric vascular graf (7).

Non-anastomotic pseudoaneurysm (PSA) is an extremely rare complication of reconstructive prosthetic vascular surgery, and consequently, only a few cases have been documented in the literature. Illuminati et al. reported a case involving an 85-year-old man who had undergone aorto—bifemoral bypass surgery approximately 20 years earlier. The patient presented with a round, pulsating mass behind the right inguinal ligament, which caused minimal pain. A confirmed diagnosis of false prosthetic aneurysm, along with a mural thrombus and associated anastomotic stenosis, was achieved through CT scan and arteriography. Microbiological examination of the prosthesis yielded negative results.

Similarly, Shiraishi et al. described a case of an 82-year-old woman, who underwent axillo-bifemoral bypass for infrarenal aortic occlusion and peripheral arterial occlusive disease 9 years before, presenting pulsatile, erythematous, indolent mass. Duplex ultrasound examination showed a leak from the bypass wall in the region of the pulsatile mass, while Computed Tomography results indicated the rupture of the left axillo-bifemoral bypass graft, leading to the formation of a non-anastomotic pseudoaneurysm (PSA). In this instance, no signs of infection were present.

Additionally, Alexandrescu et al. reported a case involving an 81-year-old man who was admitted with increasing abdominal and right inferior limb pain. A Computed Tomography-angiography (CTA) revealed a sizable 8.8 cm diameter pseudoaneurysm (PSA) filled with contrast at the origin of the right branch of the bifurcated Dacron prosthesis, detected ten years after the implantation. The pseudoaneurysm (PSA) was successfully treated with endovascular exclusion.

In all case reports considered, patients had imaging examinations when pseudoaneurysms (PSAs) became symptomatic, or evident on physical examination, which prompted the authors to emphasize the importance of periodic screening, of all patients undergoing recostructive vascular surgery, with post—operative imagings, as annual Computed Tomography-angiography (CTA) and/or Duplex Ultrasound (4, 9, 11).

## Conclusions

PSAs are an uncommon and unusual late complication of reconstructive prosthetic vascular surgery. Despite this, they are burdened with a high mortality rate and are often completely asymptomatic before they reach very significant size. Therefore, all patients undergoing reconstructive vascular surgery should be screened periodically, thus enabling early diagnosis and timely treatment.

## Data Availability

The raw data supporting the conclusions of this article will be made available by the authors, without undue reservation.

## References

[1] Wu AY Al-Jundi W Ziadi Z Barkat M Khushal A. Huge anastomotic femoral pseudoaneurysm following aorto-bifemoral bypass. BMJ Case Rep. (2011) 2011:bcr0720103160. 10.1136/bcr.07.2010.3160 22700937 PMC3079489

[2] Zhou C Langlois NE Byard RW. Femoral artery pseudoaneurysm and sudden death. J Forensic Sci. (2012) 57(1):254–6. 10.1111/j.1556-4029.2011.01897.x 21827478

[3] Peters S Braun-Dullaeus R Herold J. Pseudoaneurysm. Hamostaseologie. (2018) 38(3):166–72. 10.5482/HAMO-17-01-0006 30261523

[4] Miyake K Sakagoshi N Kitabayashi K. Transverse rupture of ring-supported dacron graft 10 years after axillobifemoral artery bypass: induced by graft deterioration and fogarty thrombectomy. J Artif Organs. (2016) 19(4):403–7. 10.1007/s10047-016-0901-1 27086125

[5] Orton DF LeVeen RF Saigh JA Culp WC Fidler JL Lynch TJ Aortic prosthetic graft infections: radiologic manifestations and implications for management. Radiographics. (2000) 20(4):977–93. 10.1148/radiographics.20.4.g00jl12977 10903688

[6] Alexandrescu V Ngongang Chr Coulon M Vandenbosschel P. Large non-anastomotic false aneurysm on dacron aortobifemoral prosthesis solved by endovascular exclusion. Acta Chir Belg. (2008) 108(6):747–9. 10.1080/00015458.2008.11680329 19241931

[7] Van Damme H Deprez M Creemers E Limet R. Intrinsic structural failure of polyester (dacron) vascular grafts. A general review. Acta Chir Belg. (2005) 105(3):249–55. 10.1080/00015458.2005.11679712 16018516

[8] Shiraishi M Kimura C Takeuchi T Muramatsu K. Late-stage non-anastomotic rupture of axillo-bifemoral bypass graft. Ann Thorac Cardiovasc Surg. (2012) 18(5):485–7. 10.5761/atcs.cr.11.01818 22446953

[9] Bensaid B Bakkali T Tijani Y Elkhalloufi S Lekehal B Sefiani Y Double localization of a non-anastomotic pseudoaneurysm after an axillofemoral bypass: a case report and review of the literature. J Med Case Rep. (2017) 11(1):3. 10.1186/s13256-016-1149-3 28049544 PMC5209889

[10] Illuminati G Bertagni A Nasti AG Montesano G. Faux anévrysme sur prothèse en dacron, vingt ans après pontage aortofémoral. Ann Chir. (2001) 126(8):783–5. 10.1016/S0003-3944(01)00597-1 11692765

[11] Shibutani S Obara H Kakefuda T Kitagawa Y. Nonanastomotic pseudoaneurysm with complete disruption of an expanded polytetrafluoroethylene axillofemoral bypass graft. Ann Vasc Surg. (2012) 26(3):422.e9–12. 10.1016/j.avsg.2011.06.017 22285346

